# A PTH-responsive circadian clock operates in *ex vivo* mouse femur fracture healing site

**DOI:** 10.1038/srep22409

**Published:** 2016-02-29

**Authors:** Tatsuya Kunimoto, Naoki Okubo, Yoichi Minami, Hiroyoshi Fujiwara, Toshihiro Hosokawa, Maki Asada, Ryo Oda, Toshikazu Kubo, Kazuhiro Yagita

**Affiliations:** 1 Department of Physiology and Systems Bioscience, Kyoto Prefectural University of Medicine, Kyoto, Japan; 2 Department of Orthopaedics, Graduate School of Medical Science, Kyoto Prefectural University of Medicine, Kyoto, Japan; 3 Department of Musculoskeletal Chronobiology, Graduate School of Medical Science, Kyoto Prefectural University of Medicine, Kyoto, Japan

## Abstract

The circadian clock contains clock genes including *Bmal1* and *Period2*, and it maintains an interval rhythm of approximately 24 hours (the circadian rhythm) in various organs including growth plate and articular cartilage. As endochondral ossification is involved not only in growth plate but also in fracture healing, we investigated the circadian clock functions in fracture sites undergoing healing. Our fracture models using external fixation involved femurs of Period2::Luciferase knock-in mice which enables the monitoring of endogenous circadian clock state via bioluminescence. Organ culture was performed by collecting femurs, and fracture sites were observed using bioluminescence imaging systems. Clear bioluminescence rhythms of 24-hour intervals were revealed in fracture healing sites. When parathyroid hormone (PTH) was administered to fractured femurs in organ culture, peak time of Period2::Luciferase activity in fracture sites and growth plates changed, indicating that PTH-responsive circadian clock functions in the mouse femur fracture healing site. While PTH is widely used in treating osteoporosis, many studies have reported that it contributes to improvement of fracture healing. Future studies of the role of this local clock in wound healing may reveal a novel function of the circadian timing mechanism in skeletal cells.

The circadian clock cycles at about 24 hours regulating physiological features including temperature, immune and endocrine systems in mammals^1^,^2^. In the mammalian clock system, the central pacemaker resides in the suprachiasmatic nuclei of the hypothalamus, coordinating peripheral cell-autonomous molecular oscillators which consist of transcription/translation feedback loops (TTFL) of clock genes, such as *Per1, Per2, Cry1, Cry2, Bmal1,* and *Clock*, performing tissue-specific functions^3^,^4^,^5^.

Among these physiological functions, previous studies have indicated that metabolism of bone and cartilage is regulated in a circadian manner^6^. It was reported that osteoblasts and osteoclasts have the circadian clock, and that bone metabolism is regulated under circadian conditions^7^,^8^,^9^. Some bone metabolic markers, such as osteocalcin, NTX or CTX, show day-night variation in human plasma or urine^10^,^11^. Clock genes such as *Per* or *Cry* influence pathways in the regulation of bone volume^12^. Moreover, chondrocytes in the growth plate and articular cartilage are also shown to have the circadian clock^14^,^15^. The growth rate of bone differs between day and night^16^. Although these findings suggest that the circadian clock plays an important role in metabolism of bone and cartilage, the exact mechanisms and contributions of a local circadian clock remain unclear.

Here, we established a mouse fracture model stabilized by external fixation. Using Period2::Luciferase (Per2::Luc) knock-in mice carrying firefly *luciferase* reporter fused with *Per2* gene^17^, we revealed robust Per2::Luc bioluminescence rhythms in a fracture healing site of the femur. Similar to bioluminescence rhythm in the growth plate or articular cartilage, the bioluminescence rhythm in the fracture healing site was reset by parathyroid hormone (PTH) treatment. These results suggest that endochondral ossification in fracture healing involves the circadian clock.

## Results

### Mouse fracture model

**Figure 1:**
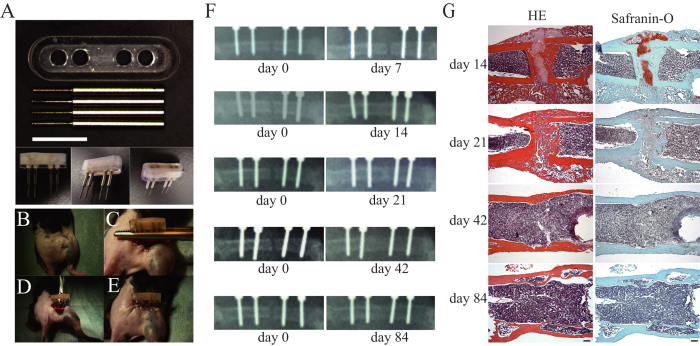
Mouse fracture model and evaluation of fracture healing. (**A**) Overview of the external fixator. White bar indicates 5 mm. (**B**–**E**) Example of the external fixator use. Details are described in Materials and Methods. (**F**) Evaluation of fracture healing by radiographs. Representative X-ray images on the day of surgery (Left) and at 7, 14, 21, 42 and 84 days after (Right). (**G**) Evaluation of fracture healing by histology. Representative results of hematoxylin and eosin (HE) stain (Left) and the safranin-O stain (Right) were shown. Femurs were collected at 14, 21, 42 and 84 days after surgery as indicated on the left side of the panels. Black bar indicates 200 μm.

Firstly, we confirmed that our mouse fracture models function adequately. Following surgery with our device (Fig. 1A), there was no abnormality in mice behavior. Callus formation was observed at the fracture site on x-ray images from post-surgical day 14 (Fig. 1F).

Histological examination of hematoxylin-eosin (HE) staining and safranin-O staining supported this finding (Fig. 1G). In femoral bones collected on post-surgical day 14, the formation of soft calli, which included cartilage tissue stained in safranin-O, was observed in the gaps of fracture sites. On post-surgical day 21, the proportion of hard calli increased, cross-bridges of woven bones were observed in the gaps between fracture sites, and the amount of cartilage tissue significantly decreased. The formation of a medullary cavity structure was observed on day 42, and an improvement of cortical structure in fracture site was observed on day 84.

### Real-time bioluminescence monitoring of Per2::Luc activity and comparative observation

**Figure 2:**
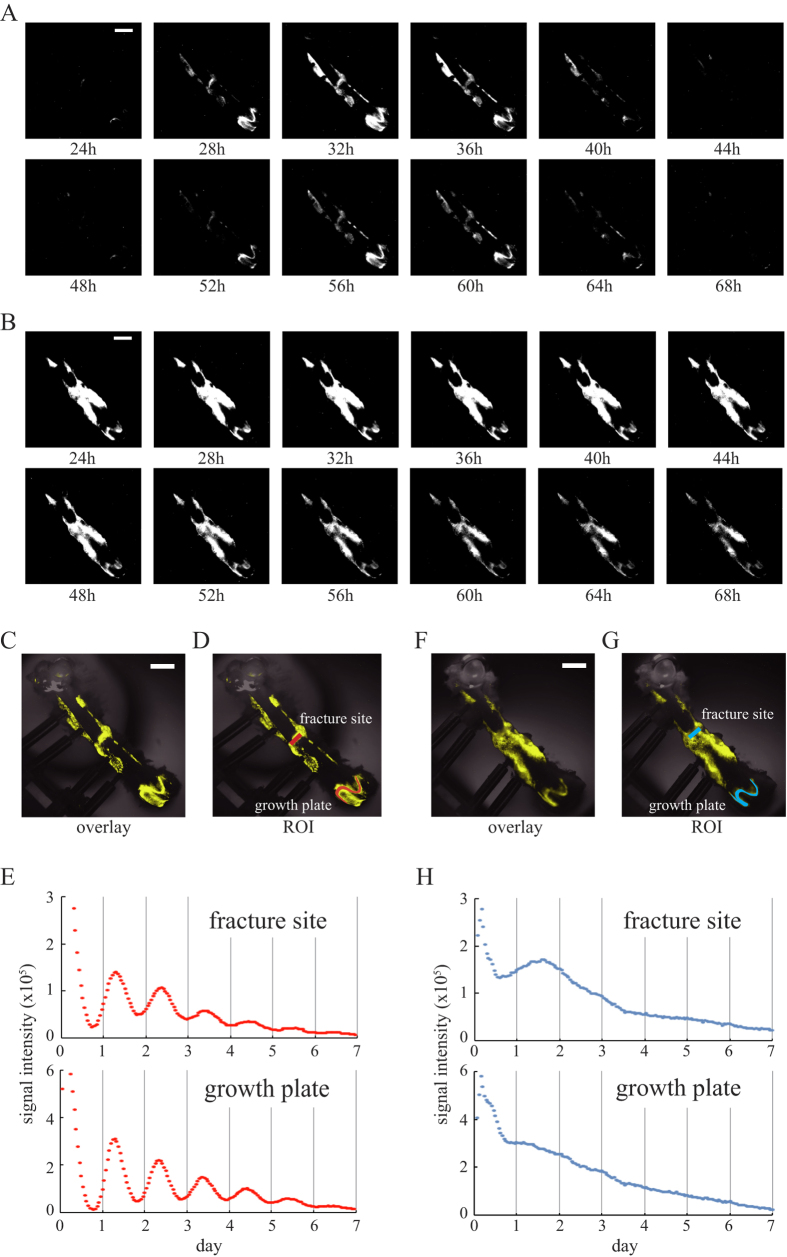
The circadian clock exists in the fracture site. Circadian rhythm of the bioluminescence from fracture femurs collected at post-surgery day 14. The time for observation start was set as time 0. (**A**) Representative bioluminescence images of Per2::Luc knock-in mouse femur obtained at 24 hours to 68 hours after measurements. (**C**,**D**) Representative overlaid image of the fracture femur of Per2::Luc knock-in mouse (**C**) and set ROIs (red) where signal intensities were measured (**D**). (**E**) Time series analysis of bioluminescence in the fracture site and the growth plate of Per2::Luc mouse. (**B**) Representative bioluminescence images of Per2::Luc, *Bmal1*^−/−^ mouse femur obtained at 24 hours to 68 hours after measurements. (**F**,**G**) Representative overlaid image of the fracture femur of Per2::Luc, *Bmal1*^−/−^ mouse (**F**) and set ROIs shown in blue (**G**,**H**) Time series analysis of bioluminescence in the fracture site and the growth plate of Per2::Luc, *Bmal1*^−/−^ mouse. To improve visibility, bioluminescence signals were displayed in yellow pseudo-color (**C,D,F,G**). White bar indicates 2 mm.

**Figure 3:**
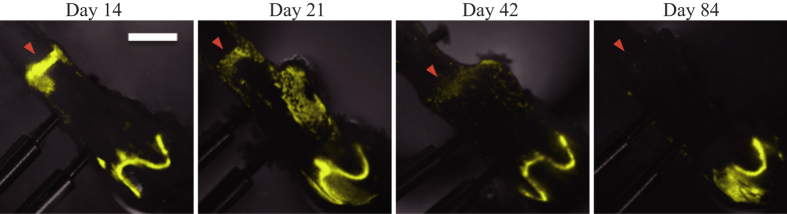
Comparative observation. Representative images obtained from femurs collected at 14, 21, 42, or 84 days after surgery. Images obtained at ZT18 were presented. Bright field image (gray scale) and bioluminescence image (yellow pseudo color) were overlaid. Arrowheads indicate fracture sites. White bar indicates 2 mm.

By using a high-sensitivity charge-coupled devices (CCD) camera-based microscopic imaging systems (ATTO, Tokyo, Japan; Olympus, Tokyo, Japan), real-time bioluminescence monitoring of Per2::Luc activity was performed with fractured femurs. Strong bioluminescence signals were observed in fracture sites, in growth plates and around the screws when the femoral bones of the Per2::Luc mice were isolated with the external fixator at post-surgical day14 (Fig. 2A,C). When bioluminescence imaging was performed every hour, clear circadian rhythms were observed in these parts for at least 6 days (Fig. 2D,E). By period analysis, period length was identified as 25.7 ± 0.61 hr (fracture site) and 25.1 ± 0.20 hr (growth plate) (n = 10, mean ± S.D.). To reveal the dependency of this oscillator on the core clock factor *Bmal1*, we performed this observation by using femurs of *Bmal1*^−/−^ mice^18^ carrying a Per2::Luc (Per2::Luc, *Bmal1*^−/−^). Although we observed strong signals in growth plates and fracture sites of femurs of Per2::Luc, *Bmal1*^−/−^ mice, the bioluminescence signals from these sites were arrhythmic (Fig. 2B,F–H). We also performed period analysis but we could not fit the data to sine wave (n = 3). In the intact femur of Per2::Luc mice, we found clear circadian oscillation of Per2::Luc activity in the growth plate and no obvious signal in the diaphyseal region (Supplemental Fig. 1). By period analysis, period length was identified as 26.1 ± 0.27 hr (n = 3, mean ± S.D.). By comparison the bioluminescence of Per2::Luc mouse femurs isolated on post-surgical days 14, 21, 42 and 84, strong bioluminescence was observed in the fracture sites, clearly identified with in femoral bones isolated on post-surgical days 14 and 21 (Fig. 3). On post-surgical day 42, however, bioluminescence in the fracture sites was weak and boundaries with the adjacent diaphyseal region became unclear. On post-surgical day 84, fracture sites could not be identified on bright field images (data not shown), and Per2::Luc signals on bioluminescence images became extremely weak (Fig. 3). We observed strong Per2::Luc activity in growth plates in all the samples of femoral bones isolated on post-surgical days 14, 21, 42 and 84.

### The effect of PTH stimulation

**Figure 4:**
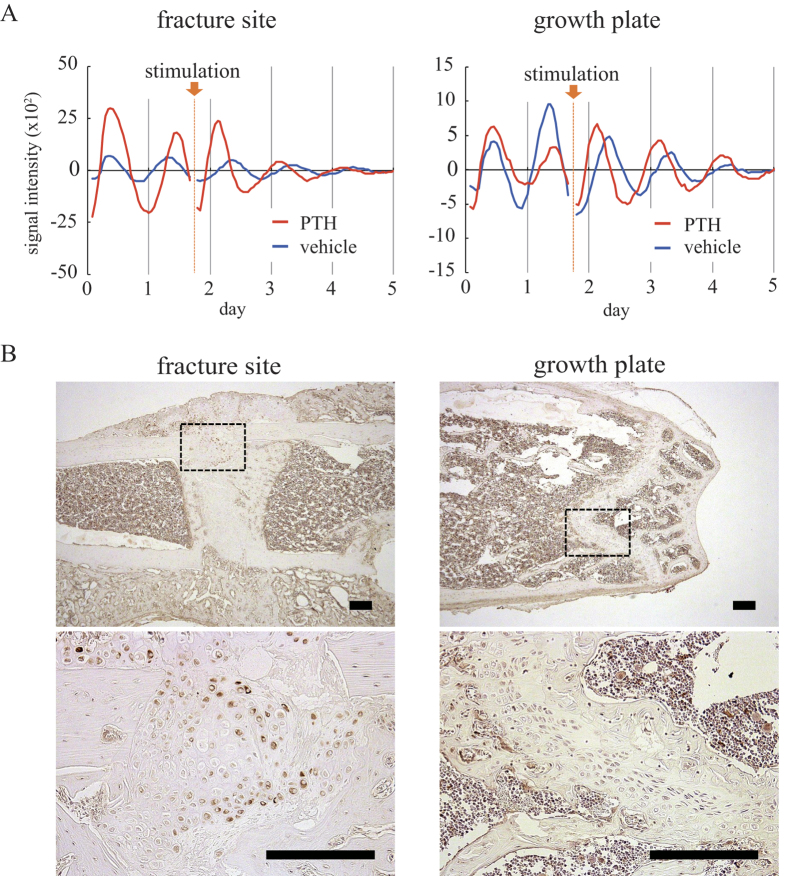
PTH effect on the circadian clock in the fracture site and growth plate. (**A**) Representative time-series analysis of the bioluminescence in the fracture sites (Left) and growth plates (Right) of the fractured femurs stimulated by PTH (red) or its vehicle (blue). We measured signal intensities in the fracture sites and growth plates. Arrow indicates PTH or vehicle stimulation. Data was detrended by subtracting the 24-hour moving average. (**B**) Immunohistochemical analysis of PTH1R. Sections of fracture site (Left) and growth plate (Right) were shown and black rectangle areas are enlarged in lower panels. Immuno-reactive cells were stained brown. Black bar indicates 200 μm.

Lastly, we studied whether PTH could reset the circadian clock in the fracture site as was observed in the growth plate^19^. Forty hours following commencement of bioluminescence rhythm observation, PTH or solvent was added to the culture medium. When solvent was added, we could observe no effect on bioluminescence rhythm in both the growth plate and the fracture site (Fig. 4A, blue lines). In contrast, with PTH stimulation, Per2::Luc activity increased in growth plates and fracture sites directly following the application of stimulation, and the next peak of bioluminescence was reached earlier than its anticipated time. Thereafter, the bioluminescence rhythm continued at approximately 24-hour intervals (Fig. 4A, red lines). We found parathyroid hormone receptor 1 (PTH1R) expression in the chondrocytes, the osteoblasts and osteocytes of fracture healing sites similar to that in chondrocytes in growth plates by immunohistochemistry (Fig. 4B).

## Discussion

In this study, we showed that molecular clocks maintain circadian rhythms in fracture healing sites by using our external fixator. In many experiments in which mouse fracture models are used, methods using internal fixation such as intramedullary nails or locking plates are frequently adopted^20^,^21^. Here, to observe fracture sites in detail, and to minimize any effects from fixators in fracture healing, we adopted an external fixation model.

By real-time monitoring of Per2::Luc activity, clear circadian rhythms were observed in both growth plates and fracture sites from Per2::Luc mice. On the other hand, Per2::Luc activity in the fracture sites and growth plates from the Per2::Luc, *Bmal1*^−/−^ mice were arrhythmic. These results indicate that *Bmal1*-dependent tissue-autonomous circadian clocks harbor not only in the growth plate but also in the fracture healing site. *Indian hedgehog* (*Ihh*), the master regulator of chondrocyte differentiation, showed circadian rhythmic expression pattern in the growth plate because its expression is directly regulated by clock genes via clock controlled element^22^. Moreover, they showed *Bmal1*^−/−^ mouse has shorter bones compared to wild-type mice in association with inhibition of *Ihh* expression^22^. Considering our result that endochondral ossification in fracture healing involves the circadian clock, circadian clock may have some role in endochondral ossification during fracture healing. Future work should perform functional wound healing studies using *Bmal1*^−/−^ mouse or other mutant models.

The intensity of bioluminescence within the fracture sites decreased and became unclear with time following surgery (Fig. 3). In a previous study, we found strong signals in cartilage parts whereas the amount of bioluminescence was extremely low in ossified bone by using a Per2::Luc mouse femur organ culture system^14^. Therefore, we consider that the decrease in the amount of bioluminescence with time following surgery was caused by a decrease in cartilage tissues. Of note, the bioluminescence signals were observed in the healing site at post-surgical day 42 when the chondrocytes were almost absent by histological analysis. Previous papers reported that both osteoblasts and osteoclasts were reported to have the circadian clock^7^,^8^,^9^. These suggest that we obtained bioluminescence signals from not only chondrocytes, but also from these other types of cells. The decrease in the amount of bioluminescence with time following surgery may be also caused by a decrease in optical translucence due to an improvement of cortical structure accompanying fracture healing.

The PTH stimulation led to a phase shift of the circadian rhythm within the bone fracture sites, similar to our previous findings that PTH resets the growth plate circadian clock^19^. Two types of PTH receptor are known; PTH1R is mainly expressed in bones and kidneys whereas PTH2R is mainly expressed in the central nervous system^23^. Hinoi *et al*. suggested that PTH activates the mouse *Per1* and *Per2* promoters through the cAMP - protein kinase A (PKA) - CRE binding protein (CREB) pathway in ATDC5 cells via PTH1R^24^. In addition, folskolin is suggested to reset the circadian clock through the cAMP - PKA - CREB pathway^25^. Our data, along with a previous report using a rat fracture healing model^26^, indicate that PTH1R is expressed in chondrocytes generated in the process of bone fracture healing. These suggest that PTH may reset the circadian clock of chondrogenic cells in the fracture healing site through the cAMP - PKA - CREB pathway via PTH1R.

While PTH agents are widely used for osteoporosis, it has been reported that they enhance the early chondrogenic stages of endochondral bone formation^27^. A previous study on the effects of teriparatide administration in osteoporosis patients reported twice the increase in bone mineral density following a morning administration compared to an evening administration^28^, and our data (this study and our previous study^19^) suggest that PTH directly changes the bone and cartilage circadian clock state. These indicate the possibility that circadian clock directly modulates PTH function of bone mineral density increase. We believe that further studies will unveil circadian clock function on fracture healing, and lead to the development of a more useful chrono-therapeutic approach of PTH.

## Methods

### Ethics Statement

The animal studies were performed with approval from the Experimental Animals Committee, Kyoto Prefectural University of Medicine (No. M25–197, M26–206). The care and use of mice were carried out in accordance with the guidelines of the Experimental Animals Committee of the Kyoto Prefectural University of Medicine.

### Experimental animals

Per2::Luc knock-in mice^17^ (12–15 week-old, males, 26.0–29.6 g), C57BL/6 mice (12–15 week-old, males, 26.4–29.5 g) were used. Surgery was performed on 19 Per2::Luc knock-in mice, and 18 were used in analysis, with 1 excluded because of infection following surgery. In creating histological samples, C57BL/6 mice purchased at 6–11 weeks old were used (SHIMIZU Laboratory Supplies Co., Ltd, Kyoto, Japan). Surgery was performed at age 12–15 weeks (n = 12). We also used Per2::Luc, *Bmal1*^−/−^ mice^18^ (12–24 weeks-old, males, 19.6–21.3 g) for bioluminescence observation (n = 3). These lines are C57BL/6 background (backcrossed over 10 generations) and maintained by intercrossing or backcrossed to C57BL/6 mice in our animal facility. We also crossed Per2::Luc mouse and *Bmal*^+/−^ mouse. To obtain Per2::Luc, *Bmal1*^−/−^ mice, we crossed male and female Per2::Luc, *Bmal1*^+/−^ mice. Genotype was confirmed by PCR method as previously reported^18^. All mice were kept in 12-hour light-dark cycles of light (7:00–19:00) and dark (19:00–7:00), respectively, with access to food and water *ad libitum.*

### External fixation and surgical procedure

In order to observe fracture sites in detail, external fixation models were used. The external fixator weighed 0.44 g including cement, acryl container and four screws (Fig. 1A). From 2 days pre- to 3 days post-surgery, analgesic agents dissolved in a water bottle (12.55 mg/500 ml tramalhydrochloride, Tramal; Nippon Shinyaku, Kyoto, Japan) were orally administered. The mice with shaved sites around the right hind leg were placed in a prone position under inhalation anesthesia with isoflurane (Fig. 1B). The operations were performed under aseptic conditions. A skin incision of approximately 12 mm was made directly above the femur from the lateral side of the right femur of the mouse. The fascia was incised, and the femur exposed by separating muscles. Following pre-drilling using a drill diameter of 0.35 mm on the frontal lateral side of the exposed femur, four stainless steel screws with a tip diameter of 0.5 mm and an axis of 1 mm (Matsumoto Industry Co., Ltd., Chiba, Japan) were inserted in parallel. After gripping the screws with forceps, an acryl container (Rihabitech, Kyoto, Japan) was set and fixed by pouring resin cement (SHOFU INC., Kyoto, Japan) into the container (Fig. 1C). After ensuring the resin cement was fully solidified, the clamping with forceps was released. An electric drill with a diameter of 0.5 mm was used in performing osteotomy in the center of the two medial screws in a direction perpendicular to the bone axis (Fig. 1D). To avoid heat effects, osteotomy was performed while cooling with phosphate-buffered solution (D-PBS(−)(1x))(Nacalai tesque, Kyoto, Japan). The wound was sutured using 5–0 nylon surgical sutures in the order of the fascia followed by the skin, and surgery was completed (Fig. 1E).

### Radiography

Directly after surgery and every 7 days thereafter, x-ray imaging of the femoral fracture sites was performed by using a Techno Mobile II (Hitachi, Tokyo, Japan), and callus formation at the fracture sites was evaluated. Imaging was performed with a lateromedial view under conditions of an effective voltage of 50 kV, a current of 100 mA, a focal distance of 100 cm and an exposure time of 0.04 seconds.

### Femur organ culture

Organ culture was performed as described by Okubo *et al*.^14^ Briefly: Per2::Luc knock-in mice were sacrificed in deep anesthesia using isoflurane for a few minutes in the evening (18:00–19:00) (Zeitgeber Time (ZT) 11–12) of each of post-surgical days 14 (n = 10), 21 (n = 4), 42 (n = 2) and 84 (n = 2). The right fractured femurs were carefully isolated with the external fixator attached. Soft tissues, including muscle tissue and tendons, were immediately removed and placed in 35 mm-diameter cell culture dishes with the culture medium^14^ and maintained at 35 °C with their lids sealed. We also cultured fractured femurs of Per2::Luc, *Bmal1*^−/−^ mice of post-surgical day 14 (n = 3). In addition, we cultured the intact right femurs of Per2::Luc mice with no surgery (n = 3) (15 week-old, males, 27.2–29.5 g). We collected these femurs at 18:00–19:00 (ZT11−12).

### Real-time bioluminescence monitoring and PTH stimulation

Fractured femurs which underwent organ culture were set in a CCD camera-based microscopic imaging systems. Bioluminescence imaging was performed every 1 hour with an exposure time of 59 minutes continuously for at least 6 days following commencement of culture. We performed quantitative analysis by Aqua Cosmos (Hamamatsu Photonics, Hamamatsu, Japan). We set the sample and started the observation at 20:00–21:00 (ZT13–14). Following at least 6 days of imaging, circadian rhythm of the bioluminescence became unclear, probably because the desynchronization of the clocks within femur^14^. Therefore, femur was exposed to forskolin (10 μM) for 1 hour to re-synchronize circadian clock^14^,^29^. Femur samples were again set with fresh culture media in the imaging devices, and bioluminescence imaging was restarted. PTH (Human, 1–34) (Peptide Institute, Inc, Ibaraki, Japan) (n = 3) or solvent (n = 3) was added 40 hours after the observation was started, and imaging was continued for a further 4 days under the same conditions. PTH dissolved in distilled water was used at a final concentration of 10^−7 ^M. Both PTH concentration and stimulation timing (41 hrs after synchronization) was determined following our previous paper^19^. In analyzing data, the 24-hour moving average was subtracted from obtained values in order to reduce trends.

### Analysis of bioluminescence data

The raw data were detrended through the subtraction of the 24 hr moving average from the raw data. To calculate the phase of the rhythms, the detrended data from 24 hr to 96 hr after synchronization were fitted to a sine wave using the least-square method. We used the following equation (1):


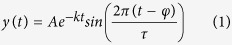


where *A* = amplitude, *k* = damping constant, *t* = time, τ = period, and φ = phase.

### HE and Safranin-O staining

C57BL/6 mice on post-surgical days 14, 21, 42 and 84 were sacrificed, their right femurs exposed, and external fixators removed. The femurs were dehydrated for 5 days using 70% ethanol following fixation for 8 hours with 4% paraformaldehyde. Thereafter, they were washed with water following decalcification for 2–4 weeks with 10% ethylenediaminetetraacetic acid (4 °C). Further, paraffin embedding was performed following immersion into 80% ethanol. Slices with a thickness of 3–4 μm in a coronal direction were created so fracture sites and growth plates would be included. Following deparaffinization, they were washed with water, and HE staining and safranin-O staining were performed (New Histo. Science Laboratory Co., Ltd, Tokyo, Japan). We also stained organ-cultured femur with HE and Safranin-O after bioluminescence observations, confirmed that the degree of fracture healing was comparable to that of the C57BL/6 femur (data not shown).

### Immunohistochemistry

Paraffin embedding was performed in the same way as in samples used for HE staining. Following deparaffinization, the samples were treated with 3% H_2_O_2_ (room temperature, 5 min) and washed with 0.01 M PBS. Rabbit polyclonal anti-PTH1R antibody (Acris Antibodies GmbH, Herford, Germany) diluted 1:150 with 0.01 M PBS was used for temporary antibodies (room temperature, 50 minutes). Histostar^TM^ mouse (Rb) (Medical & Biological Laboratories Co., LTD, Nagoya, Japan) was used for secondary antibodies, and were washed with 0.01 M PBS following reaction for 5 minutes at room temperature. After color reaction using DAB-peroxidase, nuclear staining was performed using Hematoxylin liquid. A group in which anti-rabbit IgG antibodies were used in place of primary antibodies was prepared as a negative control.

## Additional Information

**How to cite this article**: Kunimoto, T. *et al*. A PTH-responsive circadian clock operates in *ex vivo* mouse femur fracture healing site. *Sci. Rep.* **6**, 22409; doi: 10.1038/srep22409 (2016).

## Supplementary Material

Supplementary Information
