# Supplementary material for: A PTH-responsive circadian clock operates in ex vivo mouse femur fracture healing site
Source: Sci Rep. 2016 Feb 29;6:22409. doi: 10.1038/srep22409 (PMC4772627; doi:10.1038/srep22409)
Supplement: Supplementary Information [file srep22409-s1.pdf]

Supplementary information:

A PTH-responsive circadian clock operates in ex vivo mouse femur fracture healing site

Tatsuya Kunimoto[1,2,3], Naoki Okubo[1,2,3], Yoichi Minami[1,3], Hiroyoshi Fujiwara[2], Toshihiro Hosokawa[1,2,3], Maki Asada[2], Ryo Oda[2], Toshikazu Kubo[2,3], and Kazuhiro Yagita[1,*]

[1] Department of Physiology and Systems Bioscience, Kyoto Prefectural University of Medicine, Kyoto, Japan

[2] Department of Orthopaedics, Graduate School of Medical Science, Kyoto Prefectural University of Medicine, Kyoto, Japan

[3] Department of Musculoskeletal Chronobiology, Graduate School of Medical Science, Kyoto Prefectural University of Medicine, Kyoto, Japan

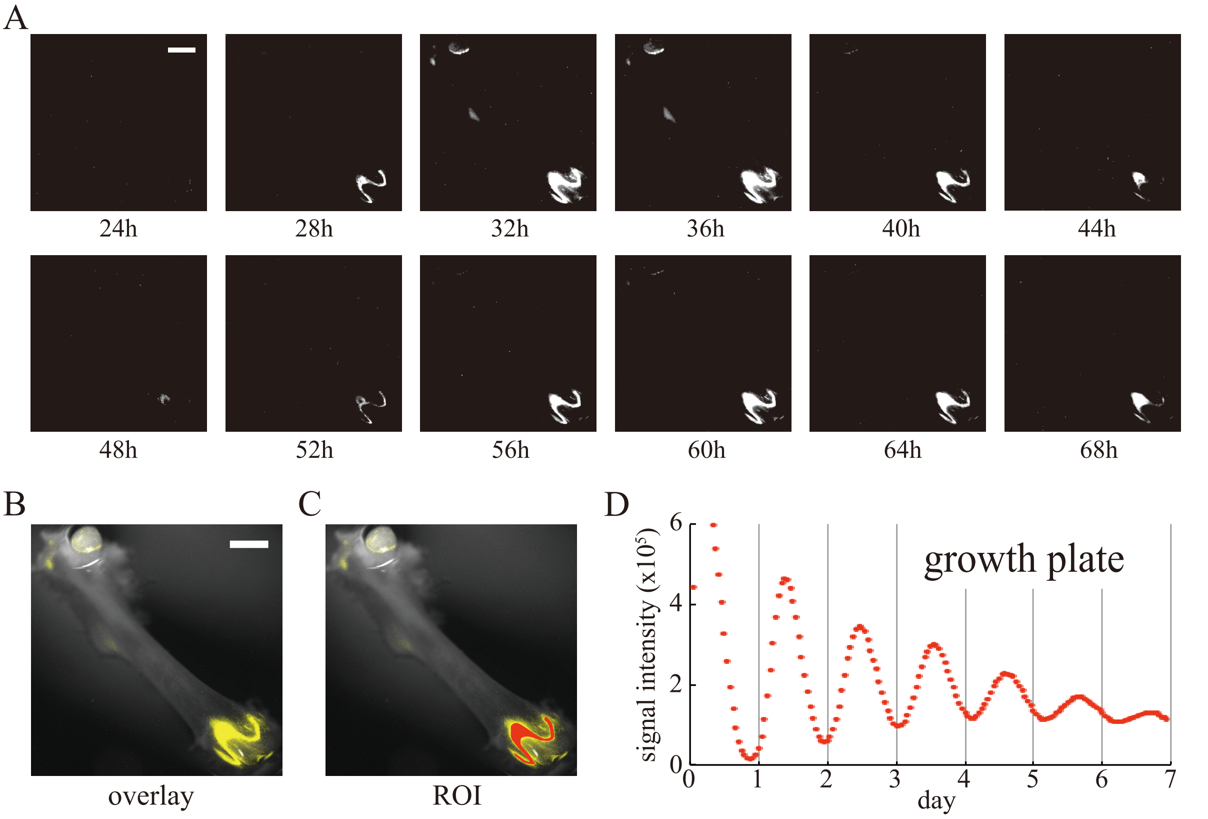

Supplemental figure 1: The circadian rhythm of the bioluminescence from growth plate of intact femur. Time 0 was set as observation starting time. (A) Representative bioluminescence images of Per2::Luc knock-in mouse femur obtained at 24 hours to 68 hours after measurements. (B, C) Representative overlaid image of femur of Per2::Luc knock-in mouse (B) and set ROIs (red) where signal intensities were measured (C). (D) Time series analysis of bioluminescence in the growth plate. To improve visibility, bioluminescence signals were displayed in yellow pseudo-color (B, C). White bar indicates 2mm.
